# Case report: Spindle cell neoplasm presenting as a spontaneous intestinal perforation in a term infant

**DOI:** 10.3389/fped.2022.952023

**Published:** 2022-08-26

**Authors:** Lauren T. Callaghan, Anthea Lafreniere, Ekene A. Onwuka, Ross M. Beckman, Jennifer H. Foster, Norma Quintanilla, Charleta Guillory, Timothy C. Lee, Lily S. Cheng

**Affiliations:** ^1^Baylor College of Medicine, Houston, TX, United States; ^2^Texas Children’s Hospital, Houston, TX, United States

**Keywords:** spindle cell neoplasm, spontaneous intestinal perforation, infantile fibrosarcoma, *BRAF*, neonatal neoplasm

## Abstract

Spontaneous intestinal perforations in the neonatal population are mostly associated with low birth weight, prematurity, and necrotizing enterocolitis. Spontaneous intestinal perforation in the absence of these risk factors is extremely rare and should raise clinical concern for an underlying bowel pathology. Here we present a unique case of a normal-weight, full-term girl with spontaneous intestinal perforation due to a spindle cell neoplasm with a novel *BRAF* mutation and infantile fibrosarcoma-like morphology. Though rare, malignancy should be considered in the differential diagnosis for bowel perforation in an otherwise healthy, term infant as complete surgical excision can be curative.

## Introduction

We present the unique case of a spontaneous intestinal perforation (SIP) in a normal-weight, term baby girl. Pathology from the bowel segment containing the perforation revealed a *BRAF*-altered spindle cell neoplasm similar in histologic appearance to infantile fibrosarcoma (IFS); however, it lacked the characteristic *ETV6:NTRK3* tyrosine kinase mutation seen in IFS (1). This rare entity of a visceral IFS-like neoplasm without the characteristic mutation has recently been recognized with only 16 cases previously reported in the literature (2–4). Our patient is the second IFS-like neoplasm with described *BRAF* gene rearrangement to present with gastrointestinal disease (5, 6).

## Case description

**FIGURE 1:**
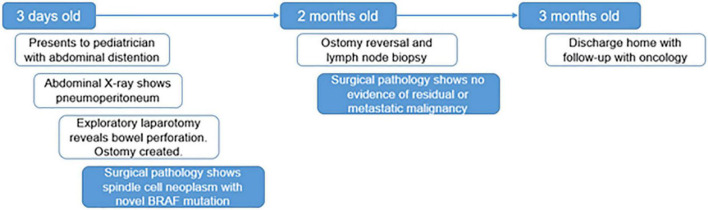
Timeline. A graphic depiction of the timeline of events in this case.

A 3-day-old, full-term baby girl presented at her first pediatrician appointment with decreased appetite, urine output, stool output, and a 14% weight loss since birth. She passed meconium on the first day of life and was breastfed. Her abdomen was noted to be distended and taut with prominent abdominal veins (Figure 1).

She had no significant prenatal history. The family history was significant only for breast cancer in a female paternal first cousin in her 4th decade of life and was otherwise unremarkable for malignancy or any intestinal diseases.

## Diagnostic assessment

**FIGURE 2:**
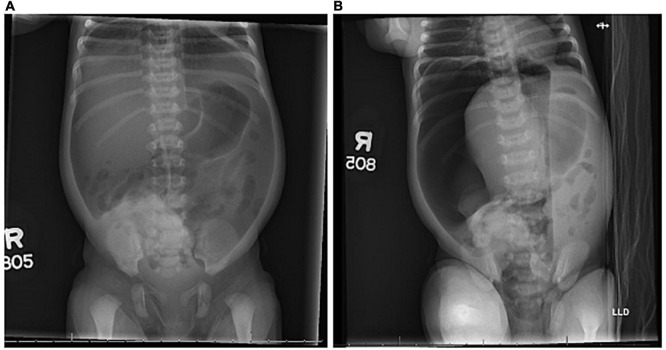
Radiographs. Supine **(A)** and left lateral decubitus **(B)** radiographs demonstrated pneumoperitoneum on day of life 3.

The patient’s pediatrician sent the patient to the emergency department where an abdominal X-ray revealed a large volume of pneumoperitoneum concerning for bowel perforation (Figure 2). Vital signs were noted to be normal with no fever. Labs were remarkable for a slight leukopenia of 4.75 (normal range: 8.04–15.40) but were otherwise unremarkable. The differential diagnosis included spontaneous intestinal perforation and necrotizing enterocolitis. A surgical consultation was immediately obtained, and the patient was emergently scheduled for abdominal exploration in the operating room.

## Therapeutic intervention

An exploratory laparotomy revealed a large perforation in the mid to distal jejunum. Purulence and friable tissue consistent with a subacute presentation of bowel perforation was noted in the abdominal cavity, but there was no evidence of necrotizing enterocolitis or any other intra-abdominal lesions. The segment of the bowel containing the perforation was resected and an enterostomy and mucus fistula were created. Given the degree of gross intra-abdominal contamination due to perforation, a primary anastomosis was not attempted.

**FIGURE 3:**
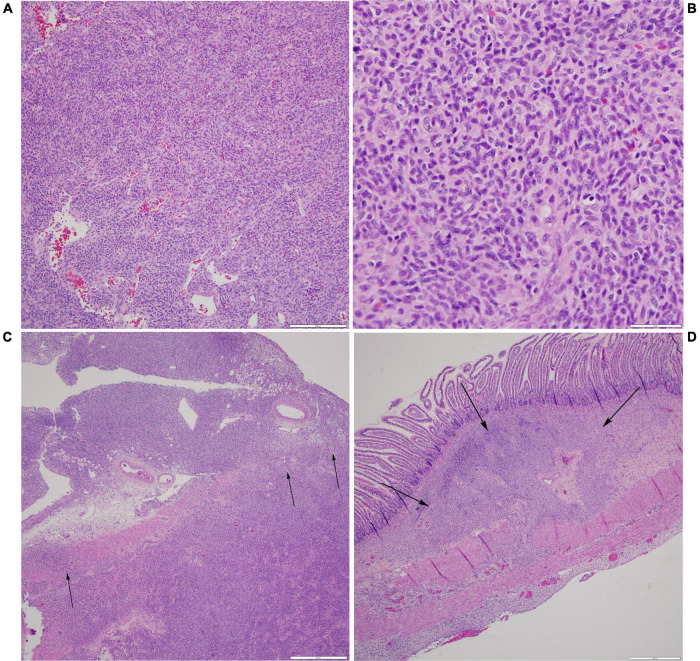
Histological examination of neoplasm. **(A)** Prominent vasculature is identified within some areas of the spindle cell neoplasm, with several vessels demonstrating a staghorn-like appearance (H&E, scale bar is 200 microns). **(B)** At higher power, the neoplastic cells are monomorphic, ovoid to spindled, with a moderate amount of cytoplasm and coarse chromatin, and scattered apoptotic bodies are noted (H&E, scale bar is 50 microns). **(C)** At the site of perforation, the neoplastic cells are seen infiltrating through the muscularis propria and into the serosa and surrounding fibroadipose tissue (H&E, scale bar is 500 microns). **(D)** These nests of neoplastic cells are away from the main site of perforation and are identified as percolating through the submucosa (H&E, scale bar is 500 microns).

Pathology of the resected bowel revealed a perforation measuring 1.4 cm × 0.8 cm in maximum diameter. There was reactive bowel wall thickening around the perforation. A microscopic tumor was identified at the site of the perforation (Figure 3). The neoplastic cells were ovoid to spindles with minimal pleomorphism, scattered mitosis (less than 1 per 10 per high power fields), and numerous apoptotic bodies. Neoplastic cells were negative for desmin, smooth muscle actin, MSA, myogenin, myoD1, beta-catenin, CD34, ERG, S100, PHOX2B, NKX2.2, pancytokeratin, CD45, CD68, CD117, ALK, CD1a, and CD207. INI-1 expression was preserved. Ki67 proliferation index was low at 1–2%. There was neoplastic infiltration of the serosa and surrounding adipose tissue, but all surgical resection margins were free of the tumor with the closest margin at 1 cm. Additionally, no lymphovascular invasion was identified.

The histopathology of the neoplasm resembled IFS; however, a single fusion gene panel did not detect the characteristic *ETV6:NTRK3* fusion transcript associated with IFS. A solid tumor comprehensive panel confirmed this result and additionally revealed two distinct mutations in *BRAF*: the first, a missense mutation of lysine to arginine in exon 15; and the second, a novel exon 1-11 variant splicing consistent with the intragenic deletion of exons 2-10. With this genetic profile, the tumor was classified as a *BRAF*-altered spindle cell neoplasm with IFS-like morphology and non-specific immunoprofile containing a novel *BRAF* mutation.

## Follow-up and outcomes

Following the diagnosis of neoplasm, a CT scan of the chest, abdomen, and pelvis was obtained to look for other sites of disease. This revealed sub-centimeter soft tissue nodules on the anterior chest and abdominal wall, but no evidence of distant disease. The patient had an ostomy reversal about 2 months after her initial laparotomy. A soft tissue nodule adjacent to the surgical incision was biopsied and showed signs of benign fibromuscular tissue with reactive changes but no malignancy. One of the radiographically identified anterior chest soft tissue nodules was biopsied at this time and pathology also revealed no evidence of malignancy or pathologic abnormality.

Based on the available literature, a primary resection with negative margins was determined to be the only necessary treatment and no adjuvant treatment was planned. The patient was followed with routine imaging and physical exams. The genetic evaluation showed no germline BRAF mutation and no BRAF-associated genetic syndromes. The patient was discharged home at 3 months of age and is currently thriving with no signs of disease recurrence.

## Discussion

Spindle cell neoplasms are a rare form of soft tissue neoplasms that were previously classified based on morphology. In the last decade, molecular oncology and immunohistochemistry have allowed this broad and unspecific notation of tumors to be subclassified into groups based on molecular signatures (7, 8). One of these pediatric subclassifications is IFS, which is most often found in the limbs of children in their first year of life (4, 9). This tumor is characterized by the molecular signature of an *ETV6:NTRK3* translocation that affects a tyrosine kinase (1, 6). While our patient’s tumor had a similar morphology to IFS, it lacked this molecular signature. However, recent studies have shown a subset of IFS-like lesions affected by related kinase mutations show a higher inclination toward visceral tumors in neonates (2–4, 10). Similar to this small subset of patients previously described in the literature, our patient presented in the few days of life with unexplained intestinal perforation. Unlike other cases previously described, our patient did not have a discrete tumor evident on laparotomy. *BRAF* gene fusion in an IFS-like neoplasm has been described in a small group of patients (5), but only once before in a visceral tumor (6).

The proto-oncogene *BRAF* codes for a serine/threonine kinase within the regulation of the MAPK/ERK signaling pathway and is vital for cell division and differentiation (11). Mutations in *BRAF* are one of the most prevalent mutations, seen in a wide variety of cancers, but only in <0.6% of soft tissue cancers (11–13). Though the large majority (80%) of *BRAF* mutation occurs at the V600 position, our patient presented with lysine to arginine substitution at the K601 position (K601N), which makes up only 7.5–11% of non-V600 *BRAF*-associated tumors. In two prior studies, this mutation has been shown to confer gain-of-function to the kinase portion of the *BRAF* gene and increases cell proliferation (11, 13, 14).

**FIGURE 4:**
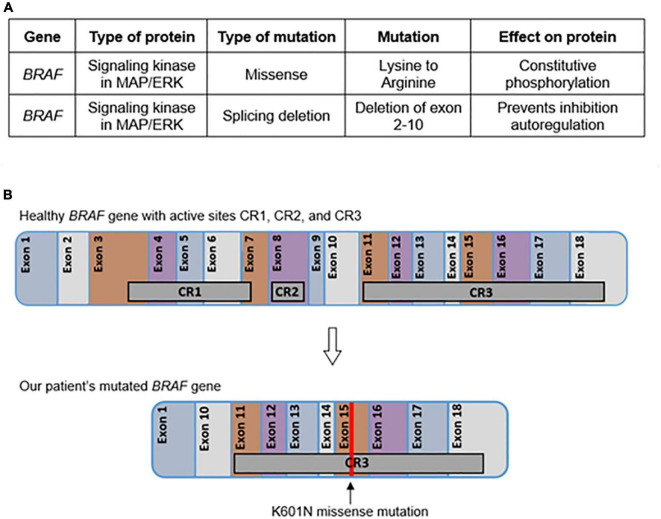
Tumor genetics. The effects of each mutation determined by the solid tumor comprehensive panel are given in table format **(A)**. The location of each active site and chromosomal change in BRAF are represented graphically **(B)**.

In addition to the K601 mutation, our patient also possessed a novel *BRAF* mutation of exon 1-11 variant splicing consistent with the intragenic deletion of exons 2-10 (Figure 4). This mutation is predicted to affect the kinase’s inhibitory autoregulation mechanism while still maintaining the ability to dimerize and activate normally (15, 16). This mutation eliminates two major conserved regions of the protein (CR1 and CR2) (Figure 4). CR1 contains the Ras binding domain (RBD) and the cysteine-rich domain (CRD) which are located in the general areas of exons 3-6 and exon 8, respectively (11). This area of the kinase has been shown *in vitro to* play a major role in the cell’s intrinsic ability to inhibit and control the activation of the MAPK/ERK pathway (17). In a functioning *BRAF* kinase, the activated Ras protein binds to the RBD and CRD to activate the kinase cascade. The splice mutation in our patient eliminates RBD and CRD and allows uninhibited activation of cascades such as the MYC, VEGF, ITGB3, and MDMD2 pathways, all of which are associated with oncogenesis. Additionally, loss of CR2 leads to loss of another route of inhibition on the *BRAF* kinase (11). This combination of mutations has not been previously described in the literature.

Mutations in *BRAF*, specifically at the K601 location, have been previously associated with a classification of germline diseases called RASopathies (17). These diseases are caused by a variety of germline pathogenic mutations within genes of the RAS/MAPK pathway and produce phenotypically similar presentations of variations of craniofacial abnormalities, epilepsy, short stature, intellectual and motor developmental delay, and cardiac abnormalities (18). Compared to other RASopathies, *BRAF*-associated cases have a higher prevalence of intellectual development delay and epilepsy. Most notably, one concerning factor of RASopathies is the higher prevalence of neoplasm, especially with mutations that are associated with oncogenesis, such as K601 (17, 18). While our patient did not present with evidence of germline *BRAF* mutation or any RASopathy-associated phenotypic features, this group of pathologies must be considered in patients presenting with a *BRAF* mutation.

Our patient’s novel *BRAF*-associated malignancy presented as SIP. Though well-documented in neonates of low birth weight, SIP is rarely seen in normal weight, full-term neonates. In the low-birth-weight neonate, intestinal perforation may occur in the presence of necrotizing enterocolitis (NEC) and is associated with high mortality and morbidity (19, 20). Risk factors include indomethacin, steroids, and maternal chorioamnionitis (21, 22). In the term neonate, intestinal perforation is associated with underlying pathologies such as Hirschsprung disease or cystic fibrosis (23, 24). SIP due to neoplasia is extremely rare in the neonatal population, yet recent case series suggest a group of intestinal IFS-type tumors may present with early, acute onset SIP in otherwise healthy infants (2–4, 10). IFS most often presents in the distal extremities in older infants, while cases affecting the gastrointestinal tract make up only 10% of cases and present mainly in neonates (3, 4, 9). Recognition of this clinical presentation is vital as our patient did not have any indication of a mass-forming lesion, which may have led to the incorrect exclusion of malignancy. Diagnosing the correct etiology of the perforation may be lifesaving as a complete surgical excision has been curative in similar cases associated with IFS or an IFS-type tumor (2, 6). Favorable prognoses have been reported with no recurrences at a 5-year follow-up (2).

Novel *ETV6:NTRK3* fusion negative, IFS-like spindle cell neoplasms have recently been recognized as a cause of early, acute onset SIP in otherwise healthy infants (2, 4). Our patient experience highlights the importance of recognizing this rare, but significant, the clinical entity in the full-term infant who presents with SIP.

## Data availability statement

The original contributions presented in this study are included in the article/supplementary material, further inquiries can be directed to the corresponding author.

## Ethics statement

Ethical review and approval was not required for the study on human participants in accordance with the local legislation and institutional requirements. Written informed consent to participate in this study was provided by the participants’ legal guardian/next of kin. Written informed consent was obtained from the minor(s)’ legal guardian/next of kin for the publication of any potentially identifiable images or data included in this article.

## Author contributions

LTC and LSC were the primary authors of the manuscript. AL and NQ contributed to the Figure 3 and provided expertise on tumor pathology and genetics. JHF provided the oncologic expertise. CG provided the expertise in neonatology. TCL provided the expertise in the surgical management of the patient. EAO and RMB contributed to the literature review. All authors reviewed and edited the final manuscript and figures.

## References

[1] Bourgeois JM Knezevich SR Mathers JA Sorensen PH. Molecular detection of the ETV6-NTRK3 gene fusion differentiates congenital fibrosarcoma from other childhood spindle cell tumors. *Am J Surg Pathol.* (2000) 24:937–46. 10895816 10.1097/00000478-200007000-00005

[2] Berrebi D Fournet JC Boman F Fabre M Philippe-Chomette P Branchereau S Intestinal congenital/infantile fibrosarcoma: a new clinico-pathological entity? *Pediatr Surg Int.* (2015) 31:375–9. 10.1007/s00383-015-3670-7 25652760

[3] Boutillier B Cardoen L Alison M Berrebi D Rosenblatt J Virlouvet AL Fatal course of abdominal neonatal intestinal fibrosarcoma. *Eur J Pediatr Surg Rep.* (2019) 7:e16–9. 10.1055/s-0039-1692154 31192106 PMC6556393

[4] Kaiser M Liegl-Atzwanger B Nagy E Sperl D Singer G Till H. Congenital infantile fibrosarcoma causing intestinal perforation in a newborn. *Case Rep Pediatr.* (2017) 2017:2969473. 10.1155/2017/2969473 28690909 PMC5485274

[5] Kao YC Fletcher CDM Alaggio R Wexler L Zhang L Sung YS Recurrent BRAF gene fusions in a subset of pediatric spindle cell sarcomas: expanding the genetic spectrum of tumors with overlapping features with infantile fibrosarcoma. *Am J Surg Pathol.* (2018) 42:28–38. 10.1097/PAS.0000000000000938 28877062 PMC5730460

[6] Penning AJ Al-Ibraheemi A Michal M Larsen BT Cho SJ Lockwood CM Novel BRAF gene fusions and activating point mutations in spindle cell sarcomas with histologic overlap with infantile fibrosarcoma. *Mod Pathol.* (2021) 34:1530–40. 10.1038/s41379-021-00806-w 33850302

[7] Jo VY Fletcher CD. WHO classification of soft tissue tumours: an update based on the 2013 (4th) edition. *Pathology.* (2014) 46:95–104. 10.1097/PAT.0000000000000050 24378391

[8] Kallen ME Hornick JL. The 2020 WHO classification: what’s new in soft tissue tumor pathology? *Am J Surg Pathol.* (2021) 45:e1–23. 32796172 10.1097/PAS.0000000000001552

[9] Suurmeijer AJH Kao YC Antonescu CR. New advances in the molecular classification of pediatric mesenchymal tumors. *Genes Chromosomes Cancer.* (2019) 58:100–10. 30187985 10.1002/gcc.22681 PMC6855396

[10] Kim HY Cho YH Byun SY Park KH. A case of congenital infantile fibrosarcoma of sigmoid colon manifesting as pneumoperitoneum in a newborn. *J Korean Med Sci.* (2013) 28:160–3. 10.3346/jkms.2013.28.1.160 23341728 PMC3546097

[11] Davies H Bignell GR Cox C Stephens P Edkins S Clegg S Mutations of the BRAF gene in human cancer. *Nature.* (2002) 417:949–54. 12068308 10.1038/nature00766

[12] Hostein I Faur N Primois C Boury F Denard J Emile JF BRAF mutation status in gastrointestinal stromal tumors. *Am J Clin Pathol.* (2010) 133:141–8. 20023270 10.1309/AJCPPCKGA2QGBJ1R

[13] Owsley J Stein MK Porter J In GK Salem M O’Day S Prevalence of class I-III BRAF mutations among 114,662 cancer patients in a large genomic database. *Exp Biol Med (Maywood).* (2021) 246:31–9. 10.1177/1535370220959657 33019809 PMC7797994

[14] Jones JC Renfro LA Al-Shamsi HO Schrock AB Rankin A Zhang BY (Non-V600) BRAF mutations define a clinically distinct molecular subtype of metastatic colorectal cancer. *J Clin Oncol.* (2017) 35:2624–30. 10.1200/JCO.2016.71.4394 28486044 PMC5549454

[15] Brummer T McInnes C. RAF kinase dimerization: implications for drug discovery and clinical outcomes. *Oncogene.* (2020) 39:4155–69. 10.1038/s41388-020-1263-y 32269299

[16] Rajakulendran T Sahmi M Lefrancois M Sicheri F Therrien M. A dimerization-dependent mechanism drives RAF catalytic activation. *Nature.* (2009) 461:542–5. 10.1038/nature08314 19727074

[17] Rauen KA. Defining RASopathy. *Dis Model Mech.* (2022) 15:dmm049344. 10.1242/dmm.049344 PMC8821523 35103797

[18] Lee Y Choi Y Seo GH Kim GH Choi IH Keum C Clinical and molecular spectra of BRAF-associated RASopathy. *J Hum Genet.* (2021) 66:389–99. 10.1038/s10038-020-00852-3 33040082

[19] Blakely ML Lally KP McDonald S Brown RL Barnhart DC Ricketts RR Network, postoperative outcomes of extremely low birth-weight infants with necrotizing enterocolitis or isolated intestinal perforation: a prospective cohort study by the NICHD Neonatal Research Network. *Ann Surg.* (2005) 241:984–9;discussion989–94. 15912048 10.1097/01.sla.0000164181.67862.7f PMC1359076

[20] Fisher JG Jones BA Gutierrez IM Hull MA Kang KH Kenny M Mortality associated with laparotomy-confirmed neonatal spontaneous intestinal perforation: a prospective 5-year multicenter analysis. *J Pediatr Surg.* (2014) 49:1215–9. 10.1016/j.jpedsurg.2013.11.051 25092079

[21] Gordon PV Attridge JT. Understanding clinical literature relevant to spontaneous intestinal perforations. *Am J Perinatol.* (2009) 26:309–16. 10.1055/s-0028-1103514 19067283

[22] Pumberger W Mayr M Kohlhauser C Weninger M. Spontaneous localized intestinal perforation in very-low-birth-weight infants: a distinct clinical entity different from necrotizing enterocolitis. *J Am Coll Surg.* (2002) 195:796–803. 12495312 10.1016/s1072-7515(02)01344-3

[23] Siddiqui MM Burge DM. Neonatal spontaneous colonic perforation due to cystic fibrosis. *Pediatr Surg Int.* (2008) 24:863–4. 18438672 10.1007/s00383-008-2164-2

[24] Zhu T Zhang G Meng X Yang J Niu Y He Y Enterocolitis is a risk factor for bowel perforation in neonates with hirschsprung’s disease: a retrospective multicenter study. *Front Pediatr.* (2022) 10:807607. 10.3389/fped.2022.807607 35198516 PMC8859433

